# Development and Usability of REACH: A Tailored Theory-Based Text Messaging Intervention for Disadvantaged Adults With Type 2 Diabetes

**DOI:** 10.2196/humanfactors.6029

**Published:** 2016-09-08

**Authors:** Lyndsay A Nelson, Lindsay S Mayberry, Kenneth Wallston, Sunil Kripalani, Erin M Bergner, Chandra Y Osborn

**Affiliations:** ^1^ Department of Medicine Vanderbilt University Medical Center Nashville, TN United States; ^2^ Center for Health Behavior and Health Education Vanderbilt University Medical Center Nashville, TN United States; ^3^ Center for Diabetes Translational Research Vanderbilt University Medical Center Nashville, TN United States; ^4^ School of Nursing Vanderbilt University Nashville, TN United States; ^5^ Center for Clinical Quality and Implementation Research Vanderbilt University Medical Center Nashville, TN United States; ^6^ Department of Biomedical Informatics Vanderbilt University Medical Center Nashville, TN United States

**Keywords:** mobile health, patient adherence, type 2 diabetes mellitus, text messaging, health status disparities

## Abstract

**Background:**

Among adults with type 2 diabetes mellitus (T2DM), adherence to recommended self-care activities is suboptimal, especially among racial and ethnic minorities with low income. Self-care nonadherence is associated with having worse glycemic control and diabetes complications. Text messaging interventions are improving the self-care of adults with T2DM, but few have been tested with disadvantaged populations.

**Objective:**

To develop Rapid Education/Encouragement And Communications for Health (REACH), a tailored, text messaging intervention to support the self-care adherence of disadvantaged patients with T2DM, based on the Information-Motivation-Behavioral skills model. We then tested REACH’s usability to make improvements before evaluating its effects.

**Methods:**

We developed REACH’s content and functionality using an empirical and theory-based approach, findings from a previously pilot-tested intervention, and the expertise of our interdisciplinary research team. We recruited 36 adults with T2DM from Federally Qualified Health Centers to participate in 1 of 3 rounds of usability testing. For 2 weeks, participants received daily text messages assessing and promoting self-care, including tailored messages addressing users’ unique barriers to adherence, and weekly text messages with adherence feedback. We analyzed quantitative and qualitative user feedback and system-collected data to improve REACH.

**Results:**

Participants were, on average, 52.4 (SD 9.5) years old, 56% (20/36) female, 63% (22/35) were a racial or ethnic minority, and 67% (22/33) had an income less than US $35,000. About half were taking insulin, and average hemoglobin A_1c_ level was 8.2% (SD 2.2%). We identified issues (eg, user concerns with message phrasing, technical restrictions with responding to assessment messages) and made improvements between testing rounds. Overall, participants favorably rated the ease of understanding (mean 9.6, SD 0.7) and helpfulness (mean 9.3, SD 1.4) of self-care promoting text messages on a scale of 1-10, responded to 96% of assessment text messages, and rated the helpfulness of feedback text messages 8.5 (SD 2.7) on a scale of 1-10. User feedback led to refining our study enrollment process so that users understood the flexibility in message timing and that computers, not people, send the messages. Furthermore, research assistants’ feedback on the enrollment process helped improve participants’ engagement with study procedures.

**Conclusions:**

Testing technology-delivered interventions with disadvantaged adults revealed preferences and concerns unique to this population. Through iterative testing and multiple data sources, we identified and responded to users’ intervention preferences, technical issues, and shortcomings in our research procedures.

## Introduction

### Overview

Currently, at least one in three people will develop type 2 diabetes mellitus (T2DM) in his or her lifetime [1]. People with diabetes are at higher risk of critical health complications including kidney failure, heart disease, and stroke [1]. More than 20% of health care spending in the United States goes toward people with a diagnosis of diabetes [1]. Racial and ethnic minorities are more likely than non-Hispanic whites to have a diagnosis of T2DM [1] and, once diagnosed, have more diabetes-related complications [2], hospitalizations [3], and premature death [4].

People with T2DM can take medication, eat healthily, exercise, and test blood glucose levels to achieve optimal glycemic control [5] and, in turn, prevent diabetes complications [6] and premature mortality [7]. However, the initiation and maintenance of these self-care activities is challenging [8], and rates of self-care adherence are low among adults with T2DM [8,9]. Adherence rates are even lower among racial and ethnic minorities [10-12] and persons of low socioeconomic status (SES) [13] owing in part to financial difficulties and misconceptions about diabetes and self-care (eg, believing they do not have control over their diabetes, believing medication is not important) [8,14-16].

Mobile phone–based interventions using text messaging are a practical approach for improving medication adherence among low-SES, racial and ethnic minorities with T2DM. More than 90% of US adults own a mobile phone [17]. Although smartphones are used less among individuals with diabetes [18], lower income [19], and lower education [19], text messaging does not require a smartphone and is the most common activity among all mobile phone users, used equally across SES, race, and ethnicity strata [20,21].

Text messaging interventions are improving the self-care and glycemic control of adults with diabetes [22-24], but few have been tested with disadvantaged populations in the United States [25]. Two prior text messaging interventions [26,27] improved glycemic control in low-SES samples but not relative to a control group. A third text messaging intervention [28] improved glycemic control among a racially diverse sample but this sample had relatively high SES. Each of these interventions use text messages to address barriers to self-care, but none identifies and addresses each user’s unique barriers. Such barriers vary from person to person with T2DM [29-32], requiring a tailored user experience.

Tailoring text messages to a user’s unique adherence barriers can address issues most applicable to him or her, such as limited diabetes knowledge, negative beliefs about medication (eg, fear of side effects), or limited financial resources [16]. Although interventions cannot easily target a person’s SES, they can enhance one’s problem-solving ability to address financial barriers and other modifiable barriers [14,16]. We developed the MEssaging for Diabetes (MED) intervention that sends tailored text messages addressing user-specific barriers to adherence and text messages assessing adherence [33]. After 3 months of MED among disadvantaged adults with T2DM, users’ barriers were reduced and barrier reduction was associated with improved glycemic control [34]. Furthermore, MED users were highly engaged, responding to 84% of daily assessment messages, and engagement did not differ by sex, race, income, health literacy, or duration of diagnosed diabetes [35]. MED’s findings are consistent with reviews suggesting text messaging interventions with personally relevant, tailored content are more engaging [36] and effective [37] than those without tailored content.

Additionally, there is mixed evidence as to whether theory-based interventions are more effective than atheoretical interventions [38,39]; this is in part due to interventions not extensively applying theory and using theories that are inappropriate for behavior change [38]. The Information-Motivation-Behavioral skills (IMB) model suggests that adherence to a behavior depends on behavior-specific knowledge, personal and social motivation, and behavioral skills [40]. The IMB model is empirically validated among a wide range of diverse samples of adults with T2DM, including samples with low SES [41-43], and explains more than 40% of the variance in their medication adherence [43]. Text messaging interventions for T2DM self-care are rarely based on health behavior theory [24,44,45]. Of the few text messaging interventions tested among disadvantaged populations with T2DM, only 2 mention using a theory-driven approach [27,28]; however, the extent to which theory was applied in these interventions is unclear.

### Objective

We developed Rapid Education/Encouragement And Communications for Health (REACH), a tailored, IMB model–based text messaging intervention. We performed 3 rounds of usability testing with adults with T2DM receiving care from Federally Qualified Health Centers (FQHCs) to identify and address any content and functionality issues before evaluating REACH’s effects on self-care and glycemic control.

## Methods

### REACH Intervention Development

REACH was developed with MEMOTEXT, an algorithmic communications and data management platform supporting personalized user outputs and inputs via short message service (SMS); interventions using this platform have been tested with diverse patient populations with different health conditions who found them acceptable, engaging, and whose adherence improved >30% [46,47]. We worked with MEMOTEXT to develop REACH based on our experience developing and testing MED [33-35].

#### REACH Content Development

Similar to MED, we created tailored text messages addressing barriers to medication adherence common in our target population; however, REACH addresses more barriers to adherence than MED and barriers map onto the IMB model. To develop REACH content, we first conducted a thorough review of published studies reporting medication adherence barriers among adults with T2DM. In October 2014, we searched for studies in PubMed using terms from each of 3 categories: (1) medication adherence (ie, diabetes medication, medication adherence, medication nonadherence, medication compliance), (2) barriers (ie, barriers, challenges, problems), and (3) diabetes or type 2 diabetes. Terms were intralinked with “OR” and “AND.” There were no restrictions on year of publication. We then searched references cited in eligible articles and articles citing relevant articles by hand. Experts in diabetes medication adherence on our team (authors SK and CYO) ensured our search captured meaningful articles. We reviewed all studies identifying barriers to diabetes medication adherence among adults diagnosed with T2DM and documented the reported barriers and race and ethnicity of the sample. Across 30 studies, we identified 68 barriers to taking medications and 7 barriers to taking insulin. We then sorted and collapsed similar barriers, resulting in 31 medication-related and 5 insulin-specific barriers. Finally, we tagged each of the 36 barriers to the IMB model’s information, motivation, or behavioral skills constructs [40], and content experts drew on identified studies to develop text messages addressing each barrier (Table 1).

Users of MED wanted text messages providing information specific to their prescribed medications. Therefore, the REACH team’s clinical pharmacist and nurse practitioner identified and classified available oral hypoglycemic agents, insulin, and noninsulin injectable drugs (glucagon-like peptide-1 receptor agonists). They then developed regimen-specific text messages on how to handle missed doses, manage medication side effects, administer medication, and store and discard medication for each class of medication.

MED users also recommended adding messages promoting other self-care behaviors (in addition to medication adherence) and inspirational messages [67]. In response, the REACH team’s dietitian/diabetes educator developed text messages with tips promoting adherence to healthful eating, physical activity, and self-monitoring of blood glucose (SMBG). These messages were developed with the goal of providing general diabetes nutrition, exercise, and SMBG statements that are applicable to people with diabetes (vs specific instructions or information that should be determined in a one-on-one consult). Therefore, guidelines for development of these messages were to generate content providing concrete and practical diabetes information applicable to most adults with T2DM. We also developed inspirational text messages to encourage the initiation and maintenance of self-care efforts (eg, “Remember that you have the power every day to make progress toward improving your health!”) and ensured all messages were contextually appropriate (eg, referenced local resources, avoided mention of things such as gym memberships).

After developing all content, the REACH team’s health communication experts reviewed and edited text messages to be readable and understandable (ie, written at the sixth-grade reading level, avoided complex terms and jargon, and health literacy appropriate). Finally, a digital content developer shortened messages (≤160 characters) and ensured consistent tone across messages and appropriateness for digital delivery.

#### REACH Functionality Development

**Figure 1:**
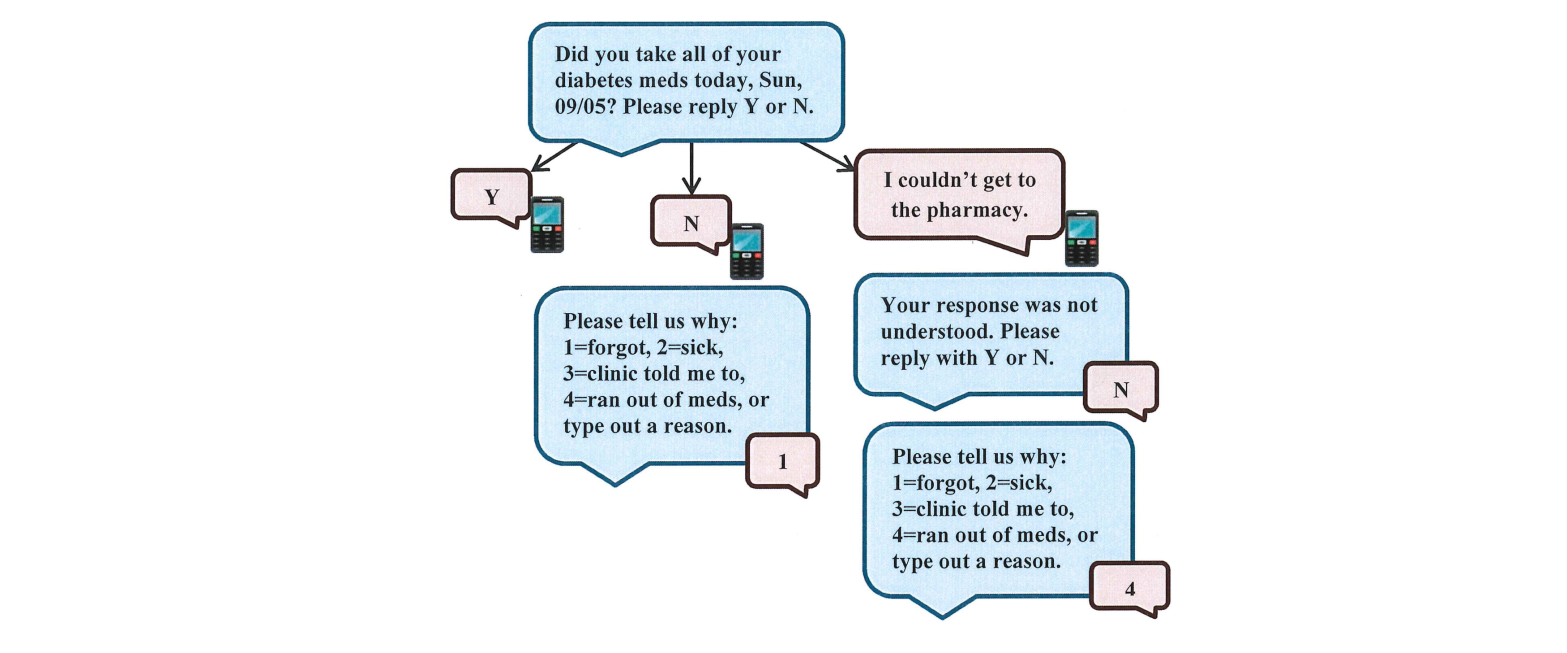
Functionality for adherence assessment text message.

With the help of MEMOTEXT, we developed functionality to optimize the REACH user experience, making it more personalized and interactive than MED. MED users received a daily text message assessing whether they took their medication that day. Users responded to this message frequently [35] and said it served as a reminder to take their medication [67]. We retained this feature in REACH but made the experience more interactive. For example, if users respond “no,” they receive a follow-up message asking why they did not take their medication with response options to encourage reflection on reasons for nonadherence (Figure 1).

MED users received feedback on their adherence (ie, aggregated responses to daily adherence assessment messages) via an interactive voice response (IVR) call. Although users enjoyed receiving adherence feedback, most said the IVR call was a nuisance [67]. They were also less likely to answer calls than respond to text messages [35]. Therefore, REACH delivers adherence feedback via a weekly text message instead of a weekly IVR call. Feedback reflects participants’ adherence for the past week and for the prior week and delivers an encouraging message tailored to whether adherence improved, declined, or stayed the same.

Finally, MED users wanted to change the times they received text messages, so REACH allows for flexibility in text message timing. Users determine a preferred window of time to receive self-care promoting text messages and indicate their bedtime for receiving adherence assessment text messages. Participants are able to adjust message timing throughout the intervention by contacting the REACH Helpline (described below).

**Table 1:** Information, Motivation, and Behavioral skills (IMB) barriers to medication adherence for patients with type 2 diabetes mellitus identified through a literature review.

Identified barriers to diabetes medication adherence		Sample	No. of text messages addressing barrier
**Information**			
	Not understanding what medication is for	AA^a^ and NHW^b^ [48]	20
	Not understanding why medication regimens change	NHW [49]	22
	Not taking medication when feeling well	AA [50,51]	16
	Seeing no immediate benefit from taking medication	Racially Diverse [52]	16
	Believing generic medication is not as good as proprietary drugs	AA and NHW [53]	16
	Believing medication is not important	AA [50]	14
	Believing it is acceptable to skip doses or stop medication	Racially Diverse [54]	16
	Believing that regularly taking medication will not help control blood glucose levels or prevent complications	Racially Diverse [52,55]	15
**Personal motivation**			
	Believing medication is harmful	AA and NHW [53]	23
	Taking medication is unpleasant	AA and NHW [48]	17
	Fear of side effects	AA and NHW [48]	20
	Worried about consequences of long-term use	Racially Diverse [55]	19
	Worried about medication causing weight gain	Racially Diverse [56]	15
	Believing that consequences of diabetes are predetermined and therefore inevitable	Racially Diverse [57]	15
	Burnout (ie, tired of taking medication)	Racially Diverse [55,56]	15
	Fear of side effects related to insulin injection^c^	Racially Diverse [58]	21
**Social motivation**			
	Not being supported by family or friends to take medications	AA [59], Racially Diverse [60,61]	16
	Help with adherence from family or friends leads to conflict.	Racially Diverse [60]	16
	Family or friends give annoying reminders to take medication	Racially Diverse [62]	17
	Feeling judged by others because you take medication	Racially Diverse [63,64]	16
	Close others are disapproving of or do not value taking medications	Racially Diverse [58]	14
	Feeling embarrassed when taking medication	Racially Diverse [58]	22
	Family priorities make it difficult to take medication regularly	AA [65], Racially Diverse [32,55]	18
	Family or friends give inaccurate information about medication	AA [65], Racially Diverse [62]	20
	Feeling judged by others because you take insulin^c^	Racially Diverse [63]	22
	Embarrassed to take insulin in public^c^	Racially Diverse [58]	13
**Behavioral skills**			
	Regimen is too complex	NHW [49], Racially Diverse [56], AA and NHW [48]	17
	Taking medication disrupts routine/life	Racially Diverse [55,57]	15
	Hard to read medication labels	Racially Diverse [31]	17
	Difficulty asking provider about medication-related problems	AA and NHW [48]	18
	Forgetting to take doses	AA [50,66], Racially Diverse [31,52,54,55,57], NHW [49], AA and NHW [48]	14
	Cost of medication	AA [50], NHW [49], Racially Diverse [31,52], AA and NHW [53]	16
	Forgetting to get refills.	AA [50]	14
	Difficulty getting refills (eg, transportation, finding a pharmacy that carries prescription and/or offers affordable options)	Racially Diverse [31,52]	19
	Not taking insulin because it interferes with daily activities^c^	Racially Diverse [58]	22
	Not knowing how to manage pain when injecting insulin^c^	Racially Diverse [58]	15

^a^AA: African American.

^b^NHW: non-Hispanic white.

^c^Only assessed among participants who were prescribed insulin.

MEMOTEXT tailors, schedules, and sends text messages using participant data received through an application programming interface (API). At enrollment, research assistants enter participants’ survey responses and electronic health record (EHR) data into REDCap, a secure, Web-based application designed to support data capture for multisite studies [68,69]. REDCap data are then transferred to MEMOTEXT via the API. MEMOTEXT tailors the messages addressing medication adherence barriers by ranking participants’ self-reported barrier scores (see Measures section) and sending messages addressing each user’s 4 highest-ranked barriers. In instances of a tie, the system randomly selects among the tied barriers. MEMOTEXT also tailors regimen-specific messages based on each user’s prescribed diabetes medication taken from the EHR.

**Figure 2:**
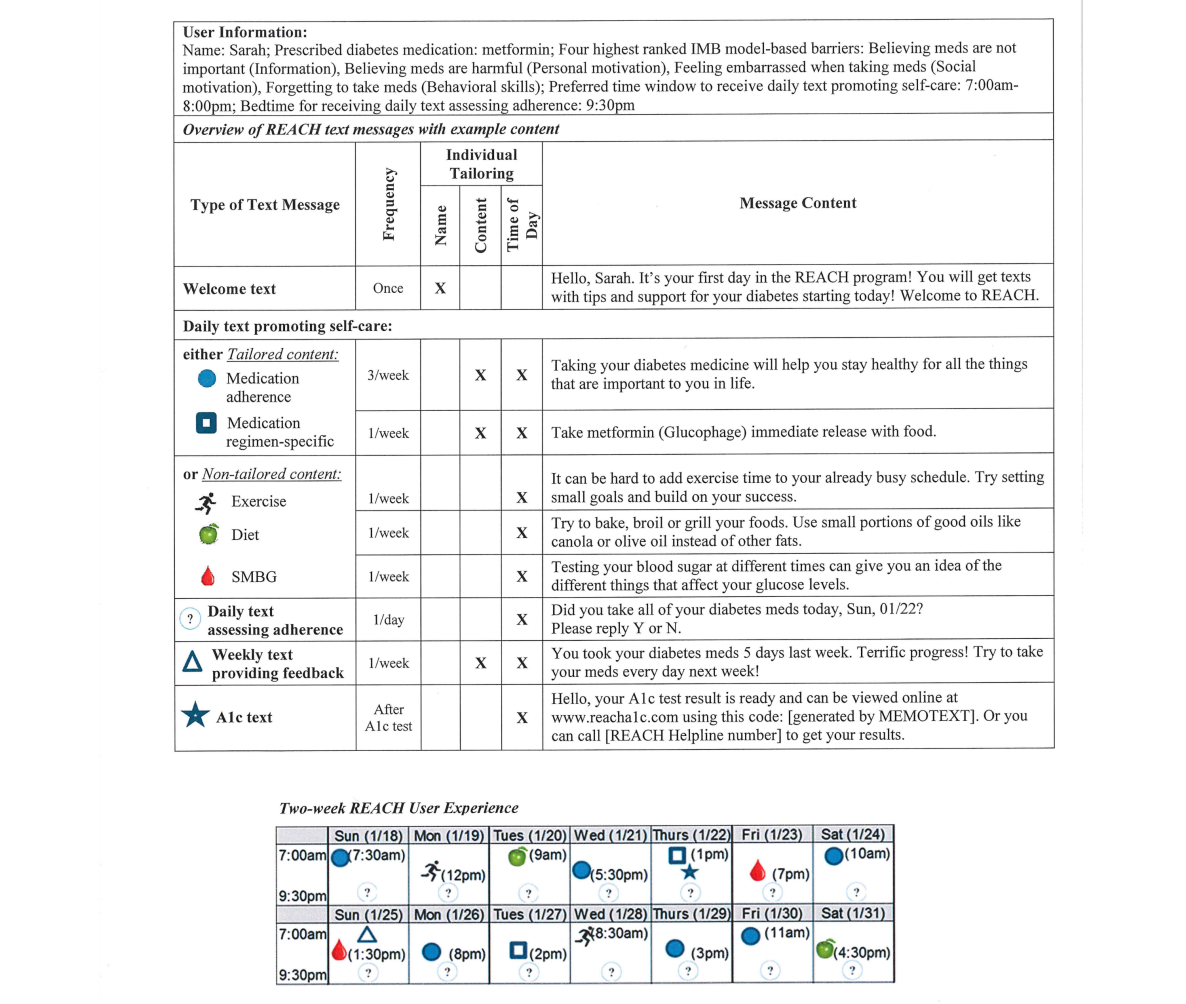
Rapid Education/Encouragement And Communications for Health (REACH) experience for a hypothetical user. Each medication adherence (blue circle) text message (3 per week) addresses one of the user’s top 4 barriers to medication adherence. IMB: information-Motivation-Behavioral skills; SMBG: self-monitoring of blood glucose; A1c: hemoglobin A1c.

We describe each REACH component in Table 2. REACH users receive 2 daily text messages: (1) a text promoting self-care—either tailored to user-identified barriers to medication adherence or nontailored to promote another self-care behavior—and (2) a text assessing medication adherence for that day. Users also receive a weekly text message with medication adherence feedback based on responses to daily assessment texts. Furthermore, after users have their hemoglobin A_1c_ (HbA_1c_) level tested during study enrollment, they receive a text message providing directions on how to access their HbA_1c_ test result; users can either log on to a Health Insurance Portability and Accountability Act (HIPAA)–compliant webpage hosted by MEMOTEXT or, if they do not have access to Internet, call the REACH Helpline. Finally, users have access to the REACH Helpline for research-, technical-, and medication-related questions. When users leave a voicemail on the Helpline, a REACH team member returns their call within one business day. Figure 2 illustrates the REACH user experience with example text messages.

**Table 2:** Rapid Education/Encouragement And Communications for Health intervention components (REACH).

Component	Description
Daily text message promoting self-care	Every day, users receive a text message promoting self-care at a random time within their prespecified window of time. Each week, REACH^a^ sends 7 of these messages, consisting of 3 tailored messages addressing 1 of their 4 identified barriers to medication adherence, 1 tailored regimen-specific message, and 3 nontailored messages providing tips for diet, exercise, or SMBG^b^ (Figure 2).
Daily text message assessing adherence	Every day, users receive a text message at their prespecified bedtime asking if they took all of their diabetes medication that day (requesting a “yes” or “no” response). Users responses may trigger follow-up messages (Figure 1).
Weekly text message delivering adherence feedback	At the end of each week, users receive a feedback text message based on the number of “yes” responses to the assessment text message for that week. The feedback is accompanied by an encouraging statement tailored to the number of days the participant adhered to their medication and whether the participant’s adherence improved, stayed the same, or declined relative to the prior week (Figure 2).
HbA_1c_^c^ text message	Participants have their HbA_1c_ level tested upon study enrollment and receive an HbA_1c_ text message when their result is ready. The HbA_1c_ text message provides directions on how to access the result, either by logging on to a HIPAA^d^-compliant webpage hosted by MEMOTEXT or calling the REACH Helpline (Figure 2).
REACH Helpline	Participants have access to the REACH Helpline, an inbound answering service hosted by MEMOTEXT. Participants call the Helpline to leave a voicemail regarding a research-related question (eg, compensation, changed phone number, accessing HbA_1c_ test result), technical question (eg, problems receiving or sending text messages), or medication-related question (eg, how to handle side effects and/or a missed dose).

^a^REACH: Rapid Education/Encouragement And Communications for Health.

^b^SMBG: self-monitoring of blood glucose.

^c^HbA_1c_: hemoglobin A_1c_.

^d^HIPAA: Health Insurance Portability and Accountability Act.

### Usability Testing

#### Sample and Recruitment

Using flyers, interest cards, and referrals from clinic staff, we recruited participants from FQHCs in Nashville, Tennessee. Eligible participants had a T2DM diagnosis, were currently prescribed at least one daily diabetes medication, were responsible for taking their diabetes medication (ie, a caregiver did not administer medication), had a mobile phone with text messaging, were at least 18 years of age, could speak and read English, and provided a social security number (necessary to process compensation). Exclusion criteria included an existing diagnosis of dementia, auditory limitations, an inability to communicate orally, and an inability to receive, read, or send a text message as determined by trained research assistants.

#### Data and Procedures

The Vanderbilt University Institutional Review Board approved all study procedures before enrollment. Research assistants met with interested patients to describe the study and verify eligibility. In a private room at the patient’s clinic, research assistants administered a brief cognitive screening instrument [70] and sent a test text message to each patient to assess whether he or she could see, read, and successfully respond to the message. If a patient passed this screener, research assistants obtained informed consent before verbally administering survey measures. A clinic phlebotomist performed a blood-drawn HbA_1c_ test. Research assistants accessed participants’ EHRs to confirm a T2DM diagnosis, collect the type and quantity of prescribed diabetes medication, and the study HbA_1c_ test result.

During each testing round, participants experienced REACH for 2 weeks and then completed a semistructured phone interview that qualitatively assessed their user experience. Following each round, research assistants collected user feedback, the REACH team resolved content- and research-related issues, and MEMOTEXT resolved technical issues before the next round. Participants received up to US $54 for completing the enrollment survey (US $20), replying to assessment messages (US $1/day), and completing the phone interview (US $20).

#### Measures

##### Sample Characteristics

We collected self-reported age, sex, race, ethnicity, income, education (ie, years in school), insulin status, and diabetes duration (ie, years since a diabetes diagnosis). We also asked about comfort with mobile phones and text messaging and used validated survey instruments to capture additional information.

##### Barriers to Medication Adherence

Respondents rated how much each barrier in Table 1 (written as statements, eg, “I’m not sure what my diabetes medicine is supposed to do”) gets in the way of taking their diabetes medication from 1=“not at all” to 10=“a lot.” Each item maps onto a single IMB model–based barrier.

##### REACH Engagement

We measured engagement with system-collected responses to daily adherence assessment texts, the frequency of REACH Helpline calls, and the frequency of accessing HbA_1c_ test results via the website. We calculated engagement with assessment text messages by dividing each participant’s number of responses by the total number of messages sent to him or her. MEMOTEXT tracked Helpline calls and HbA_1c_ website use.

##### User Feedback

Likert-type items assessed ease of understanding and helpfulness of the REACH intervention elements. Open-ended items assessed what users did and/or did not like, asked how and why an element was or was not helpful, and elicited suggestions for improving the REACH user experience. Table 3 presents items used to elicit user feedback.

#### Analyses

We calculated descriptive statistics with SPSS Statistics version 23 (IBM Corp). Interviews were audio-recorded and transcribed verbatim by an external transcription service. Questions and responses were pasted into REDCap under each interview question, organized by testing round, and then exported to Excel. We undertook a pragmatic approach to analyze participant feedback quickly between rounds to support changes to the intervention in a timely fashion. Between rounds, a member of the research team (LAN) read interview transcripts to manually categorize participants’ feedback by intervention component. We then looked across participants’ comments for each intervention component to identify areas for improvement and to ascertain the overall tone and message of the users’ feedback for each component.

**Table 3:** User feedback interview items by intervention element.

Element	Item format	Item content	Mean (SD)
Daily self-care text message	Likert scale	On a scale from 1-10, where 1 is not easy and 10 is 1 very easy, how easy was it for you to understand the messages that gave tips?	9.6 (0.7)
		On a scale from 1-10, where 1 is not helpful and 10 is very helpful, how helpful were those messages to you?	9.3 (1.4)
	Open-ended	Can you tell me why you chose that number? (Follow-up to question above.)	N/A^a^
		Tell me about some of the messages you received that were very helpful. Why were those messages helpful?	N/A
		Tell me about some messages that did not help you or did not apply to you. Why did the messages not help or apply to you?	N/A
Daily assessment text message	Likert scale	On scale from 1-10, where 1 is not helpful and 10 is very helpful, how helpful were those messages to you?	9.1 (2.1)
	Open-ended	Can you tell me why you chose that number? (Follow-up to question above.)	N/A
		Is there anything else you can tell me about your experience with the text messages that asked if you took your meds?	N/A
Weekly adherence feedback text message	Likert scale	On a scale from 1 to 10, where 1 is not at all 1 and 10 is very much, how much did the messages at the end of the week help you take care of your diabetes?	8.5 (2.7)
	Open-ended	Can you tell me why you chose that number? (Follow-up to question above.)	N/A
		Is there anything else you can tell me about your experience with the text messages that asked if you took your meds?	N/A
Hemoglobin A_1c_ text message	Open-ended	Why did you/did you not access your A1c^b^ result using information in the text message?	N/A
		What are your thoughts about receiving your A1c test result online or by calling our research team?	N/A
REACH^c^ helpline	Open-ended	Why did you/did you not use the Helpline?	N/A

^a^N/A: not applicable.

^b^A1c: hemoglobin A_1c_.

^c^REACH: Rapid Education/Encouragement And Communications for Health.

## Results

### Participant characteristics

An average of 12 participants experienced REACH each testing round, totaling 36 participants (Table 4). The average age of the participants was 52.4 (SD 9.5) years, 63% (22/35) were a racial or ethnic minority, 39% (14/36) had less than a high school degree or equivalent, and 67% (22/33) had an income less than US $35,000. The average HbA_1c_ level was 8.2% (SD 2.2%); 64% (23/36) of the participants had suboptimal glycemic control (HbA_1c_≥7.0%). Across rounds, the most frequently reported barriers to medication adherence were forgetting to take doses (56%, 20/36 users reported this barrier with an average score of 5.2, SD 3.0, of 10), the high cost of medication (44%, 16/36 users; mean score 6.2, SD 2.7, of 10), and believing that taking medication is unpleasant (42%, 15/36 users; mean score 5.2, SD 2.6, of 10). The most commonly reported insulin-specific barrier was feeling embarrassed to take insulin in public (35%, 6/17 users who were prescribed insulin; mean score 4.5, SD 2.4, of 10).

**Table 4:** Participant characteristics.

Characteristics		Total (N=36)	Iterative testing round		
			1 (n=10)	2 (n=13)	3 (n=13)
Age in years, mean (SD)		52.4 (9.5)	51.6 (9.1)	52.4 (11.7)	52.8 (7.8)
**Sex, n (%)**					
	Male	16 (44.4)	6 (60.0)	4 (30.8)	6 (46.2)
	Female	20 (55.6)	4 (40.0)	9 (69.2)	7 (53.8)
**Race** ^a^ **, n (%)**					
	White	13 (37.1)	3 (30.0)	5 (38.5)	5 (41.7)
	Nonwhite^b^	22 (62.8)	7 (70.0)	8 (61.5)	7 (58.3)
Education, years, mean (SD)		13.7 (2.5)	14.0 (3.0)	13.8 (2.3)	13.3 (2.4)
**Annual household income** ^c^ **, US$, n (%)**					
	<10,000	7 (21.2)	1 (12.5)	4 (30.8)	2 (16.7)
	10,000-34,999	15 (45.4)	3 (37.5)	6 (46.2)	6 (50.0)
	≥35,000	11 (33.3)	4 (50.0)	3 (23.1)	4 (33.3)
Comfortable with using mobile phone, n (%)		36 (100.0)	10 (100.0)	13 (100.0)	13 (100.0)
Text message with mobile phone, n (%)		36 (100.0)	10 (100.0)	13 (100.0)	13 (100.0)
Diabetes duration, years, mean (SD)		7.3 (6.0)	7.4 (6.5)	9.4 (6.4)	5.0 (4.5)
Number of prescribed diabetes medications, mean (SD)		1.7 (0.8)	1.9 (0.7)	1.8 (1.0)	1.4 (0.8)
Insulin status, taking insulin, n (%)		17 (47.2)	4 (40.0)	7 (53.8)	6 (46.2)
**Health literacy (BHLS** ^d^ **), mean (SD)**		11.4 (2.7)	10.9 (3.1)	11.6 (2.2)	11.6 (2.9)
	Limited (≤9), n (%)	10 (27.8)	3 (30)	3 (23.1)	4 (30.8)
	Adequate (>9), n (%)	26 (72.2)	7 (70)	10 (76.9)	9 (69.2)
Medication adherence (ARMS-D^e^), mean (SD)		25.4 (2.9)	25.2 (2.6)	25.5 (2.0)	25.4 (4.0)
General diet (SDSCA^f^), mean (SD)		3.8 (1.9)	4.4 (2.0)	3.8 (1.4)	3.4 (2.3)
Specific diet (SDSCA), mean (SD)		3.6 (1.4)	3.7 (1.9)	3.5 (1.0)	3.7 (1.4)
Exercise (SDSCA), mean (SD)		2.6 (2.5)	4.4 (2.3)	2.2 (2.4)	1.7 (2.1)
SMBG^g^ (SDSCA), mean (SD)		3.0 (2.8)	3.4 (2.9)	3.3 (2.6)	2.4 (2.9)
Glycemic control (HbA_1c_^h^, %), mean (SD)		8.2 (2.2)	9.3 (2.8)	8.1 (1.9)	7.5 (1.9)

^a^One participant did not report race.

^b^Nonwhite participants were majority (77.3% (17/22)) African American.

^c^A total of 3 participants did not report annual household income.

^d^BHLS: Brief Health Literacy Screen.

^e^ARMS-D: Adherence to Refills and Medications Scale for Diabetes (possible range 7-28).

^f^SDSCA: Summary of Diabetes Self-Care Activities (number of days with medication adherence in the past week).

^g^SMBG: self-monitoring of blood glucose.

^h^HbA_1c_: hemoglobin A_1c_.

All 36 participants completed an exit interview. Overall, participants said REACH was helpful and gave favorable feedback on each intervention element. Participants reported preferences and technical issues requiring iterative improvements between testing rounds. Below, we describe this feedback, our iterative changes by intervention element, followed by changes in our research processes.

### Daily Text Message Promoting Self-Care

Across rounds, participants rated the ease of understanding and helpfulness of the daily self-care text message, on average, 9.6 (SD 0.7) and 9.3 (SD 1.4) on a scale of 1-10, respectively. Participants appreciated that these messages were simple and without medical jargon. Participants said inspirational messages made them feel supported and not alone in living with diabetes and motivated them to take more initiative with self-care. Messages with self-care tips and information were helpful because they either provided a useful reminder or communicated something new.

Interviewer: *Why did you read those messages? What made you want to?*

Participant: *They was [sic] helpful. Some things I didn't know [sic]. They helped me understand a lot of stuff because I didn't understand. [37-year-old, African American male]*

Many participants valued reminders to care for their diabetes when they otherwise might not think about it:

*I thought these messages were very helpful. I get so busy in the day that I don’t [even] take time to eat. And then when I get a text, [I realize] oh, wow, I need to do something. That really helps a lot. I wish I had somebody who did that for me all the time. [59-year-old, white male]*

Despite the overall positive feedback about self-care messages, some participants had concerns. For example, a round 2 participant said a message provided a suggestion for remembering to take medications, without providing the steps for carrying out the suggestion. A few participants said some messages implied a problem when they did not have one (eg, “Struggling to take your diabetes medications every day? Talk to a loved one about what is getting in your way.”). To address such concerns, we revised all problematic text messages between rounds 2 and 3 to provide additional context and be less presumptuous (see Table 5 for examples of problematic and revised text messages).

### Daily Text Message Assessing Adherence

Across rounds, participants responded to 96% of adherence assessment text messages and, on average, rated the helpfulness of these messages 9.1 (SD 2.1) out of 10. Assessment messages helped remind participants to take their medications and maintain their routine. One participant commented on these messages’ emotional and social support:

*[The texts] keep you on task about what you should do...especially if someone doesn't have anybody around. You know it's kind of like having a family member around to remind you, “Hey, you should take your meds.” These [texts] make you feel like someone cares or is concerned about your health and makes sure you're taking care of yourself. So I think that's very helpful. [48-year-old, African American male]*

Several participants with optimal glycemic control (HbA_1c_ <7%) said these messages were not particularly helpful because they routinely took their medication and rarely missed doses. Nonetheless, these participants endorsed the value of these messages for others recently diagnosed with diabetes and/or newly prescribed medication who do not have an established routine.

Round 1 participants complained about needing to respond to assessment messages several times before the system would accept their response. Upon viewing system-collected data, we learned that participants used different variations of “Yes” to respond (eg, “Yup” or “Yeah”). Therefore, we expanded the acceptable response options representing “Yes” and “No” between rounds (see Table 5).

### Weekly Text Message Providing Feedback

Across rounds, participants rated the helpfulness of the weekly feedback messages, on average, 8.5 (SD 2.7) out of 10. Round 1 participants had two concerns with the weekly adherence feedback text message. First, many participants felt these messages were wordy and confusing. Because the message provided numerical information about the number of days a participant took his or her medication in both the past and the prior week, the content was difficult to read and interpret. We simplified feedback messages by including only the number of adherent days from the past week, but we indicated whether adherence had improved, stayed the same, or declined with an encouraging statement (Table 5).

Second, some round 1 participants complained their feedback underreported their adherence. This was due, in part, to the limited number of accepted responses to the assessment message (described above). However, we also learned the system was not counting responses received after midnight on the day it sent this message, so we extended the response time window (Table 5). In round 1, participants rated the helpfulness of feedback messages 8.2 (SD 3.0) out of 10. After revising feedback text messages and resolving functionality issues, participants in subsequent rounds rated the helpfulness of these messages 8.8 (SD 2.2) out of 10.

### Hemoglobin A1c Text Message

Across rounds, very few participants accessed their HbA_1c_ test result. One participant logged into the HbA_1c_ website and 2 participants called the REACH Helpline to get the result over the phone. Participants’ most common reason for not using either option was that they learned their HbA_1c_ test result from their clinic before receiving the HbA_1c_ text message. When asked their opinion about accessing their result with the HbA_1c_ text message, some participants appreciated this convenience, whereas others preferred their health care provider contact them with the result. On the basis of this feedback and the feedback from providers who preferred delivering and individually interpreting HbA_1c_ test results, we reduced our interpretation of the HbA_1c_ test result on the website (Table 5) and over the phone.

**Table 5:** Changes made to the Rapid Education/Encouragement And Communications for Health intervention during usability testing (REACH).

Type of change		Example or description
**Content**		
	Revising daily text messages promoting self-care	Rounds 1 and 2: “Sometimes you can see stress coming. When this happens, make a plan for how to keep up your diabetes med routine during the storm.” “Ask any pharmacist for help coming up with a daily plan. Together, you may be able to group your meds into a few set times each day.” Round 3: “If you look at your calendar and can see a busy, stressful week ahead, make a plan now for how to keep up with your med routine during the chaos.” “If you’re struggling to come up with a daily plan for your meds, ask your pharmacist for help. He or she can help you group them into a few set times each day.”
	Revising weekly adherence feedback text messages	Round 1: “Congrats! You took all of your diabetes meds on 3 day(s) last week, which is better than 2 day(s) the prior week. Keep up the good work!” Rounds 2 and 3: “You took your diabetes meds 3 days last week. You’re making progress, but keep working to take your meds every day!”
	Revising HbA_1c_^a^ test result interpretation provided on HbA_1c_ webpage and over phone	Round 1: 6%-7%: This is within the normal range for a person with diabetes. Great job. Keep up the good work! 7.1%-8.9%: This is a little above the goal range. It is often recommended patients be as close to 7 as their nurse or doctor recommends. You may want to discuss this with someone at your next clinic appointment. 9% and above: This number is above where we want our patients to typically be. You may want to discuss this with someone at your next clinic appointment. Rounds 2 and 3: 7% or lower: at goal 7.1% to 8.9%: high 9% or higher: very high If you have any questions, please contact your doctor.
**Functional**		
	Expanding acceptable responses for assessment text message	Round 1 response options: “Y,” “Yes,” “N,” “No.” Rounds 2 and 3 response options: “Yes,” “Y,” “Yea,” “Yeah,” “Ya,” “Yep,” “Yup,” “No,” “N,” “Nope,” “Na,” and if any of these responses are included at the beginning of a response (eg, “yes, ma’am”; “no, ma’am”).
	Extending window for assessment text message responses	Round 1: system would only accept responses to assessment messages sent by midnight of the night an assessment message was received. Rounds 2 and 3: system accepts responses to assessment messages until a message promoting self-care is received the following day.
**Research processes**		
	Creating a two-stage process for barrier assessment	Round 1: participants rated how much each barrier got in the way of taking their diabetes medication on a scale from 1 = “not at all” to 10 = “a lot.” Rounds 2 and 3: First, participants sort cards with each barrier printed on them into piles labeled “Never” or “Sometimes” based on whether the barrier applies to them. Next, participants rate the degree to which each barrier placed in the “Sometimes” pile applies to them from 1 = “a little” to 10 = “a lot.”
	Modifying instructions provided during enrollment process	Round 1: Many participants were unaware that they could change the timing of their messages and that text messages were automated. Rounds 2 and 3: Research assistants provided explicit instruction during enrollment process of the flexibility in message timing and how to change timing at any point. Additionally, we included language in the informed consent document that indicated a computer system was sending text messages and responses were not being monitored.

^a^HbA_1c_: hemoglobin A_1c_.

### REACH Helpline

We received 22 voice mails on the REACH Helpline (12 research-related, 8 technical-related, and 2 medication-related voice mails) from 11 participants (8 of whom called more than once). When we asked the other 25 participants about the Helpline, most said they simply did not need it but thought they might use it if the program lasted longer.

### Research Processes

On the basis of participant feedback and lessons learned by research staff, we made several changes to our research process. One change involved modifying how we administered the barrier assessment. Initially, research assistants asked participants to rate how much each of the barrier items gets in the way of taking their medication by reading each item aloud sequentially and asking for a rating. After round 1, research assistants reported some participants became disinterested/disengaged when completing this assessment and 20% reported no barriers despite having suboptimal HbA_1c_ levels. Therefore, after round 1, we changed the barrier assessment to a two-stage process. The first stage is a card-sorting task in which participants sort cards with barrier statements printed on them (see Measures section) into piles labeled “Sometimes” or “Never” based on whether or not the barrier applies to them. Next, research assistants ask participants to rate the degree to which each barrier placed in the “Sometimes” pile applies to them from 1=“a little” to 10=“a lot.” Before the two-stage process, round 1 participants reported a total of 62 barriers. After implementing the two-stage process, round 2 and round 3 participants reported 87 and 92 barriers, respectively. According to research assistants, participants in rounds 2 and 3 were more engaged during the barrier assessment process than participants in round 1.

We also modified the instructions provided during enrollment. Round 1 participants did not know they could change the timing of their text messages, so, in subsequent rounds, we clarified that participants could call the REACH Helpline at any point to request a time change. Also, during round 1, many participants sent unprompted responses (eg, “Thanks” or “OK, I will”) to the text messages promoting self-care, suggesting they thought a person sent these messages. Therefore, we revised our informed consent to make clear that a computer was sending text messages and not a person. For additional safeguarding, MEMOTEXT monitors all text message responses and notifies the REACH team if any text message requires follow-up.

## Discussion

### Principal Findings

Text messaging interventions provide an opportune platform for extending the delivery of tailored diabetes education and support; however, few have been designed for and tested among disadvantaged persons with T2DM [27,28]. We developed REACH—a tailored text messaging intervention designed to overcome user-specific medication adherence barriers and support other self-care behaviors—and tested its usability among patients with T2DM who were representative of the population REACH is designed for (ie, racially diverse, low SES, more than 25% limited health literacy). Participants who experienced REACH for 2 weeks had favorable opinions and responded frequently to daily text messages. We learned participants’ concerns/preferences, technical issues, and problems with our research process that we then fixed between each testing round, improving REACH in preparation for an evaluative trial.

Overall, participants were satisfied with REACH and provided favorable ratings for each of its elements. Text messages provided emotional/social support, reminded participants to engage in self-care activities, and helped them keep their self-care routine on track. In a similar 4-week study, Dick et al [71] assessed the usability of a text messaging program (SMS-DMCare) for improving T2DM self-care among African Americans. Participants provided ratings comparable to REACH regarding SMS-DMCare’s ease of use and provided similar interview feedback (eg, messages were helpful by reminding participants to take medication amid the demands of their daily lives) [71]. REACH’s text message engagement was higher than SMS-DMCare’s engagement [71], which may be due to REACH’s tailored content and/or personalized adherence feedback.

Usability studies often rely on survey- or questionnaire-based feedback [72], which may overlook much of what participants like or do not like about a system and how to improve it. Georgsson and Staggers [73] endorse using multiple data sources to identify and address usability issues. We collected both quantitative and qualitative feedback and system-collected data to fully understand users’ experience, improve our programmatic content and functionality, and resolve technical problems. REACH users who were adults from racially diverse and low-SES groups expressed concerns with the phrasing and wordiness of some messages, so we improved them. Furthermore, unanticipated feedback from research assistants and clinic staff was instrumental in refining our research process.

Through this multiple data source approach to usability testing, we made several improvements to REACH and enriched the user experience. We revised text messages to be more comprehensive, clear, and consistent with participants’ preferences. We limited our interpretation of HbA_1c_ test results to be more respectful of provider-patient relationships. We also improved REACH’s functionality to ensure the system recognizes and records participants’ attempts to interact with the intervention. Finally, we improved our assessment for capturing participants’ adherence barriers and modified our informed consent to ensure participants know how to use each intervention component and that a computer, not a person, sends all text messages.

There are several limitations to this study. First, participants experienced REACH for 2 weeks. Therefore, feedback and engagement may not be representative of participants experiencing REACH for longer periods. Furthermore, we compensated participants US $54 for their participation in the 2-week usability testing and feedback interview. By providing this incentive, we sought to adequately compensate participants for time and travel to the enrollment appointment and to offset mobile phone costs associated with text messages and the phone interview. This compensation may have inflated engagement with the intervention, but it was important for usability testing that participants actually use the intervention to be able to provide meaningful feedback. Despite this compensation, few participants accessed the REACH Helpline and HbA_1c_ website, making it difficult to gain insight on how participants felt about these elements and their functionality. Furthermore, participants may have been reluctant to provide critical feedback owing to study compensation, social desirability, or associating the study with their clinic. Additionally, because we were interested in specific questions concerning each intervention element, we composed our own feedback interview items and do not have validity and reliability information to report. Finally, although our sample size far exceeded the targeted enrollment for qualitative (at least 5) and quantitative (at least 20) usability testing [74,75], our sample was still too small to examine differences in opinions by participant characteristics.

### Conclusions

Usability testing is imperative for ensuring that effects identified during efficacy trials are due to the intervention as intended and not due to errors in understanding or using the intervention [72]. Moreover, involving disadvantaged adults in usability testing may reveal preferences and concerns unique to this population. Iterative usability testing of the REACH intervention using multiple data sources revealed shortcomings in content, functionality, and research processes that we addressed before evaluating its effects on adherence and glycemic control in a randomized controlled trial. The REACH randomized controlled trial will assess the intervention’s effectiveness by recruiting patients from FQHCs and comparing outcomes between patients who did and did not experience REACH. To gain knowledge about REACH’s potential for implementation in clinic settings as a supplement to usual care, we will compensate participants for completing study assessments but will not provide them mobile phones or mobile phone plans. Although users experienced and commented on REACH specifically, our usability testing process and findings are applicable to other technology-delivered health interventions for disadvantaged populations. Specifically, informed consent should affirm that participants understand an intervention’s functionality, capabilities, and automation. Additionally, intervention content should provide enough information to be useful and avoid implying that a user is experiencing a specific issue; this is challenging while maintaining brevity necessary for digital content. Finally, our findings emphasize the importance of using multiple methods and sources of data to identify and resolve usability issues.

## Abbreviations

EHR: electronic health record
FQHC: Federally Qualified Health Center
HbA_1c_: hemoglobin A1c
HIPAA: Health Insurance Portability and Accountability Act
IMB: information-motivation-behavioral skills
IVR: interactive voice response
MED: MEssaging for Diabetes
REACH: Rapid Education/Encouragement And Communications for Health
SES: socioeconomic status
SMBG: self-monitoring of blood glucose
SMS: short message service
T2DM: type 2 diabetes mellitus
